# Critical roles of SMYD2-mediated β-catenin methylation for nuclear translocation and activation of Wnt signaling

**DOI:** 10.18632/oncotarget.19646

**Published:** 2017-07-27

**Authors:** Xiaolan Deng, Ryuji Hamamoto, Theodore Vougiouklakis, Rui Wang, Yuichiro Yoshioka, Takehiro Suzuki, Naoshi Dohmae, Yo Matsuo, Jae-Hyun Park, Yusuke Nakamura

**Affiliations:** ^1^ Department of Medicine, The University of Chicago, Chicago, IL, USA; ^2^ Department of Surgery, The University of Chicago, Chicago, IL, USA; ^3^ Biomolecular Characterization Unit, RIKEN Center for Sustainable Resource Science, Wako, Saitama, Japan; ^4^ OncoTherapy Science, Inc., Kawasaki, Kanagawa, Japan

**Keywords:** β-catenin, Wnt signaling pathway, SMYD2, lysine methylation

## Abstract

Accumulation of β-catenin in the nucleus is a hallmark of activation of the Wnt/β-catenin signaling pathway, which drives development of a large proportion of human cancers. However, the mechanism of β-catenin nuclear translocation has not been well investigated. Here we report biological significance of SMYD2-mediated lysine 133 (K133) methylation of β-catenin on its nuclear translocation. Knockdown of SMYD2 attenuates the nuclear localization of β-catenin protein in human cancer cells. Consequently, transcriptional levels of well-known Wnt-signaling molecules, *cMYC* and *CCND1*, are significantly reduced. Substitution of lysine 133 to alanine in β-catenin almost completely abolishes its nuclear localization. We also demonstrate the K133 methylation is critical for the interaction of β-catenin with FOXM1. Furthermore, after treatment with a SMYD2 inhibitor, significant reduction of nuclear β-catenin and subsequent induction of cancer cell death are observed. Accordingly, our results imply that β-catenin methylation by SMYD2 promotes its nuclear translocation and activation of Wnt signaling.

## INTRODUCTION

Wnt/β-catenin pathway is a canonical Wnt signaling pathway that is critical for stem cell regulation and is also known to be activated in a large proportion of human cancers such as colorectal cancer and hepatocellular carcinoma (HCC). β-catenin functions as the transcriptional co-activator in the Wnt signaling pathway and transmits the signal by regulating the downstream genes including key developmental genes or those involved in development/progression of human cancer [1–5]. Nuclear β-catenin is considered as a primary transducer of the canonical Wnt signaling in the nucleus and to represent the hallmark for activation of the Wnt/β-catenin pathway [4, 5]. Although it is well known that dysfunction of molecules involved in the β-catenin-degradation machinery such as APC, Axin, or GSK3β as well as β-catenin activating mutations cause the accumulation of β-catenin in the nucleus, the molecular mechanisms of its nuclear translocation has not been well investigated.

SMYD2 was first identified as one of the SMYD family members and functions as an oncogene, which is highly expressed in various types of human cancer. Our group has reported critical roles of SMYD2-mediated methylation in tumorigenesis as a methyltransferase [6–9].

Since SMYD2 protein is mainly located in the cytoplasm and overexpressed frequently in colorectal and liver cancers where the Wnt/β-catenin pathway is also frequently activated, we aimed to investigate a possible relationship between SMYD2 and β-catenin.

Here we report biological significance of SMYD2-mediated lysine 133 (K133) methylation of β-catenin on its nuclear translocation. We show the K133 methylation is critical for the interaction of β-catenin with FOXM1. After treatment with a SMYD2 inhibitor, significant reduction of nuclear β-catenin and subsequent induction of cancer cell death are observed. Our results imply that β-catenin methylation by SMYD2 promotes its nuclear translocation and activation of Wnt signaling.

## RESULTS

### SMYD2 methylates β-catenin *in vitro* and *in vivo*

**Figure 1:**
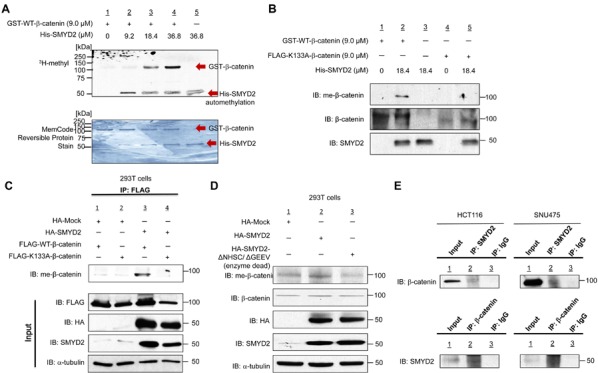
SMYD2 methylates β-catenin *in vitro* and *in vivo* **A.** Recombinant GST-WT-β-catenin protein (118 kDa) was methylated by His-SMYD2 (50 kDa) in a dose-dependent manner. Human recombinant GST-WT-β-catenin protein and S-adenosyl-L-methionine (SAM) were incubated in the absence or presence of recombinant His-SMYD2 protein. Methylated β-catenin was detected by fluorography, and amounts of loading proteins were evaluated by MemCode^TM^ Reversible Protein Stain. **B.** *In vitro* validation of SMYD2-mediated β-catenin methylation using anti-monomethylated K133 β-catenin antibody (meK133-β-catenin). Human recombinant GST-WT-β-catenin protein, FLAG-K133A-β-catenin protein and S-adenosyl-L-methionine (SAM) were incubated in the absence or presence of recombinant His-SMYD2. Samples were immunoblotted with the anti-meK133-β-catenin antibody. Methylated β-catenin was detected in the presence of His-SMYD2 protein, while no methylation band could be detected in FLAG-K133A-β-catenin. **C.** Detection of methylated β-catenin in 293T cells. 293T cells were transfected with a FLAG-WT-β-catenin vector or a FLAG-K133A-substituted β-catenin vector together with HA-Mock or HA-SMYD2 vector, followed by immunoprecipitation with anti-FLAG M2 agarose. Samples were immunoblotted with anti-meK133-β-catenin antibody after immunoprecipitation, and with anti-FLAG, anti-HA, anti-SMYD2 and anti-α-tubulin antibody before immunoprecipitation (input). **D.** Detection of methylated β-catenin in 293T cells. Cells were transfected with HA-Mock, HA-SMYD2 or enzyme-dead HA-SMYD2 (ΔNHSC/ ΔGEEV), followed by immunoblotting with anti-meK133-β-catenin, anti-β-catenin, anti-HA, anti-SMYD2 and anti-α -tubulin antibodies. **E.** Endogenous interaction of β-catenin and SMYD2 in HCT116 and SNU475 cell lines. Cell extracts of HCT116 and SNU475 were subjected to immunoprecipitation using anti-SMYD2 antibody or IgG, followed by immunoblotting with anti-β-catenin antibody (upper panels). Reciprocal immunoprecipitation was done using anti-β-catenin antibody or control IgG, followed by immunoblotting with anti-SMYD2 antibody (lower panels).

We first performed an *in vitro* methyltransferase assay using recombinant His-SMYD2 protein and recombinant GST-WT (wild type)-β-catenin to examine a possibility whether β-catenin can be a potential substrate of SMYD2, and found that SMYD2 could methylate β-catenin in a dose-dependent manner (Figure 1A). We subsequently performed liquid chromatography-tandem mass spectrometry (LC-MS/MS) analysis to define a methylation site(s) in β-catenin by SMYD2 and identified that lysine 133 (K133) was monomethylated by SMYD2 (Supplementary Figure 1). We subsequently generated an anti-K133 monomethylated β-catenin antibody and confirmed its high specificity against the K133-methylated peptide by an ELISA assay (Supplementary Figure 2). Then we further validated SMYD2-mediated β-catenin methylation by western blot analysis using this antibody after an *in vitro* methyltransferase assay (Figure 1B). A K133-methylated β-catenin band was observed in the presence of SMYD2 protein, while no positive band was seen in the absence of SMYD2, further supporting that lysine 133 in β-catenin is methylated by SMYD2. We also constructed a recombinant plasmid to express K133A (a lysine residue is replaced to an alanine residue)-substituted β-catenin protein and then performed an *in vitro* methyltransferase assay. Expectedly, K133A-substituted β-catenin protein could not be methylated by SMYD2, supporting that lysine 133 is the SMYD2-mediated methylation site in β-catenin (Figure 1B).

Furthermore, we transfected a vector construct expressing FLAG-tagged wild-type (WT)- or K133A-substituted-β-catenin, with HA-SMYD2 vector into 293T cells. Then we immunoprecipitated cell lysates with the anti-FLAG antibody and performed western blot analysis using the anti-K133-methylated β-catenin antibody (Figure 1C). The antibody recognized WT-β-catenin but could not recognize K133A-substituted β-catenin, confirming monomethylation of K133 by SMYD2. We also conducted the western blot analysis after transfection of HA-mock, HA-SMYD2 or enzyme-dead HA-SMYD2 (ΔNHSC/ΔGEEV) vector into 293T cells. The methylation level of endogenous β-catenin was significantly increased in the cells in which HA-SMYD2 was exogenously introduced. On the other hand, the cells with enzyme-dead HA-SMYD2 showed the lowest level of methylated β-catenin; this might reflect the dominant-negative effect of enzyme-dead-HA-SMYD2 on endogenous SMYD2 in 293T cells (Figure 1D) (This enzyme-dead-HA-SMYD2 could probably interact with β-catenin, but could not methylate it). In addition, we confirmed the interaction between endogenous SMYD2 and β-catenin by co-immunoprecipitation assays in two cancer cell lines, HCT116 (colon cancer) and SNU475 (hepatocellular carcinoma (HCC)) (Figure 1E). Taken together, these results indicate that SMYD2 methylates β-catenin at lysine 133 both *in vitro* and *in vivo*.

### SMYD2-mediated methylation of β-catenin plays a critical role on nuclear translocation of β-catenin

Since it is known that the majority of SMYD2 protein is located in the cytoplasm, we hypothesized that methylation of a lysine residue in β-catenin might affect its subcellular localization in cancer cells as we reported examples in our review [10, 11] and in turn, regulate the Wnt signaling pathway. Since the activation of Wnt signaling caused by β-catenin, APC or Axin1 mutation is a common event in the great majority of colon cancer and in a subset of HCC [12, 13], we chose four cancer cell lines for subsequent experiments; two colon cancer cell lines, HCT116 and SW480, harboring *β-catenin* mutation and *APC* mutation, respectively, as well as two HCC cell lines, SNU449 and SNU475, with *β-catenin* mutation and *Axin1* mutation, respectively.

**Figure 2:**
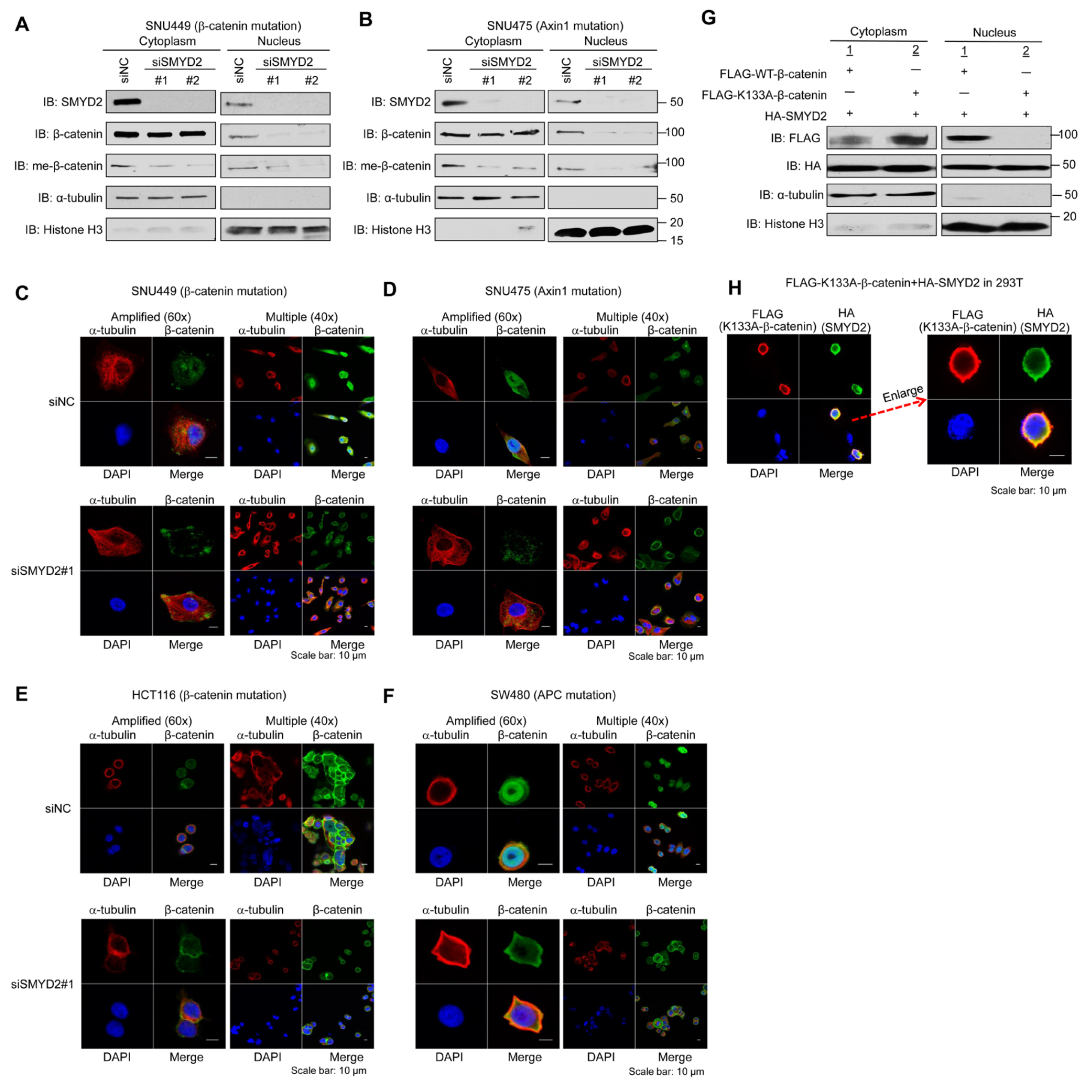
SMYD2-mediated methylation of β-catenin plays a critical role on nuclear translocation of β-catenin (A, B) Effect of SMYD2 knockdown on the amount of nuclear β-catenin and that of K133-methylated endogenous β-catenin in cytoplasm and nucleus of SNU449 **A.** and SNU475 **B.** cells by western blot analysis. Cells were transfected with control siRNA (siNC) or either of two SMYD2-specific siRNAs (siSMYD2-#1 and -#2). 72 h after siRNA transfection, nuclear and cytoplasmic fractions were prepared and immunoblotted with anti-SMYD2, anti-β-catenin, anti-meK133-β-catenin, anti-α -tubulin and anti-histone H3 antibodies. (C-F) Disappearance or significant reduction of nuclear β-catenin by SMYD2 knockdown was observed in SNU449 **C.**, SNU475 **D.**, HCT116 **E.** and SW480 **F.** cells by ICC analysis. Cells were transfected with siNC (control) or siSMYD2 (SMYD2#1). After 48h-incubation with siRNA for SNU449 and SNU475 cells, or after 24h-incubation for HCT116 and SW480 cells, cells were fixed with 4% paraformaldehyde, and stained with an anti-α -tubulin antibody (Alexa Fluor^®^ 594, red), anti-β-catenin antibody (Alexa Fluor^®^ 488, green) and 4′,6′-diamidine-2′-phenylindole dihydrochloride (DAPI, blue). (G, H) Lack of nuclear β-catenin after introduction of methylation-deficient (K133A) β-catenin was observed by western blot analysis **G.** and ICC analysis **H.**. 293T cells were transfected with FLAG-WT-β-catenin or FLAG-K133A-substituted β-catenin, together with HA-SMYD2. For western blot analysis, nuclear and cytoplasmic fractions were prepared from the cells after 48h-incubation and immunoblotted with anti-FLAG, anti-HA, anti-α -tubulin and anti-histone H3 antibodies. For ICC analysis, after 48 h of incubation, the cells were fixed and stained with an anti-FLAG (K133A-substituted β-catenin) antibody (mouse, Alexa Fluor^®^594, red), an anti-HA (SMYD2) antibody (rat, Alexa Fluor^®^ 488, green) and 4′,6′-diamidine-2′-phenylindole dihydrochloride (DAPI, blue).

We knocked down SMYD2 in SNU449 and SNU475 cells using two *SMYD2*-specific siRNAs (siSMYD2-#1, and -#2), and conducted western blot analysis after fractioning cytoplasmic and nuclear components. We found that nuclear β-catenin as well as endogenously methylated β-catenin (both in the cytoplasm and nucleus) in these two cell lines was significantly decreased by SMYD2 knockdown (Figure 2A, 2B). Subsequent immunocytochemical analysis (ICC) after SMYD2 knockdown confirmed drastic reduction of nuclear β-catenin in these two cell lines (Figure 2C, 2D), while β-catenin nuclear accumulation was clearly observed in the cells treated with control siRNA (siNC). Additional ICC analysis using a specific antibody for monomethylated β-catenin on K133 further showed reduction of monomethylated β-catenin in the nucleus of cells after SMYD2 knockdown (Supplementary Figure 3). For HCT116 and SW480 colon cancer cell lines, in which *β-catenin* or *APC* is mutated [14–16], ICC experiments (Figure 2E, 2F) revealed similar phenotypic changes showing that SMYD2 knockdown strikingly decreased the amount of nuclear β-catenin, implying that SMYD2-mediated β-catenin methylation is essential for β-catenin nuclear translocation regardless to the dysfunction in the β-catenin ubiquitination machinery or β-catenin activation by the β-catenin mutation. Among 100 cells we examined, 98 %, 97 % and 84 % of SNU449, SNU475 and HCT116 cells, respectively, revealed disappearance of nuclear β-catenin in the SMYD2 knockdown group while almost all cells in the control group showed accumulation of high levels of nuclear β-catenin. However, the effect of SMYD2 knockdown on nuclear β-catenin in SW480 cells was relatively modest (Figure 2F); only 30% of the cells revealed the loss of nuclear β-catenin.

To further confirm the significance of SMYD2-mediated β-catenin methylation for its nuclear translocation, we co-transfected WT-β-catenin vector or K133A-substituted β-catenin vector with a plasmid designed to express HA-SMYD2 into 293T cells. Western blot analysis of lysates prepared from the cytoplasmic and nuclear components of the cells detected a large amount of WT-β-catenin in the nucleus, while nuclear β-catenin was almost completely diminished when we used K133A-substituted β-catenin construct (Figure 2G), further supporting that K133-methylation in β-catenin is critically essential for the nuclear translocation of β-catenin protein. The lack of nuclear localization of K133A-substituted β-catenin was also confirmed by ICC analysis (Figure 2H). Furthermore, we examined the effect of exogenous introduction of SMYD2 on nuclear level of endogenous β-catenin under Wnt3a-stimulated condition using 293T cells (Supplementary Figure 4). In the presence of exogenous SMYD2, the level of endogenous β-catenin in the nucleus in 293T cells was significantly higher than the basal level of Wnt3a-stimulated condition. By co-expression of HA-tagged exogenous SMYD2 (WT or enzyme-dead) and FLAG-tagged exogenous β-catenin (WT or K133A-substitution) in 293T cells, we observed nuclear β-catenin only in the cells co-transfected with WT-β-catenin and WT-SMYD2, but not in those expressing SMYD2 enzyme-dead or K133A -β-catenin (Supplementary Figure 5), strongly supporting that the nuclear localization of β-catenin is dependent on the enzyme activity of SMYD2 that monomethylates the K133 residue in β-catenin.

### SMYD2-mediated methylation is required for β-catenin nuclear translocation and activation of Wnt downstream genes

**Figure 3:**
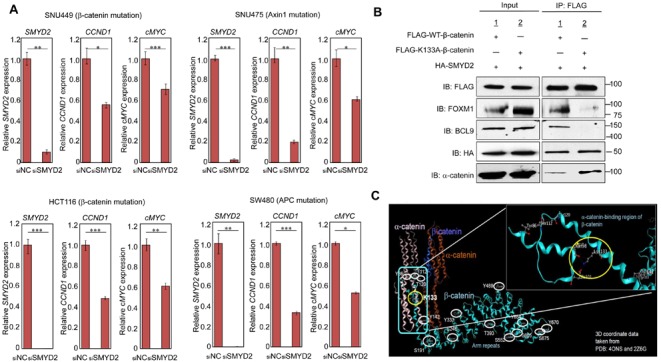
SMYD2-mediated β-catenin methylation is required for expression of Wnt downstream genes and β-catenin nuclear translocation by FOXM1 **A.** SMYD2 knockdown attenuated transcriptional levels of two pivotal Wnt pathway downstream genes, *CCND1* and c*MYC* in two HCC cell lines, SNU449 (upper left panel ) and SNU475 (upper right panel ), and two colon cancer cell lines, HCT116 (lower left panel) and SW480 cells (lower right panel). Cells were transfected with siNC or siSMYD2 (siSMYD2#1). After 48h-incubation, RNAs were prepared from these cells and transcriptional levels of *SMYD2*, *CCND1* and c*MYC* were measured by quantitative RT-PCR. Statistical analyses were performed using unpaired Student's *t*-test (two groups). The asterisks indicate statistical significance; *, **, and *** indicate *p*-value of < 0.05, < 0.01 and < 0.005, respectively, compared to the corresponding value of the siNC (control) group. Error bars indicate values of one standard deviation (*n* = 3). **B.** SMYD2-mediated methylation at K133 of β-catenin is required for its interaction with FOXM1, BCL9 and α -catenin. 293T cells were co-transfected with FLAG-WT-β-catenin or FLAG-K133A-substituted β-catenin, and HA-SMYD2. After 48-h incubation, cell lysates were immunoblotted with anti-FLAG (WT-β-catenin or K133A-substituted β-catenin), anti-FOXM1, anti-BCL9, anti-α -catenin, and anti-HA (SMYD2) antibodies before immunoprecipitation (input), and after immunoprecipitation with anti-FLAG M2 agarose. FOXM1 and BCL9 were co-immunoprecipitated with FLAG-WT-β-catenin, but not with FLAG-K133A-substituted β-catenin. α -catenin was co-immunoprecipitated more preferentially with FLAG-K133A-substituted β-catenin than FLAG-WT-β-catenin. However, HA-SMYD2 was co-immunoprecipitated almost equally with both WT- and K133A-substituted β-catenin proteins. **C.** Structural analysis of an α -catenin-binding region of β-catenin including lysine 133. Without methylation of lysine 133 of β-catenin, hydrogen bonds are made between lysine 133 (K133) and methionine 98 (M98), and between lysine 133 and glutamic acid 101 (Glu 101) in an α -catenin-binding region of β-catenin. When K133 is methylated, these hydrogen bonds might be affected and the affinity of β-catenin to α -catenin could be reduced. The crystal structure of residues 84-682 of β-catenin is obtained from PDB: 4ONS and 2Z6G.

To investigate the importance of β-catenin methylation in regulation of the Wnt/β-catenin/TCF pathway, we performed quantitative RT-PCR to examine mRNA levels of some pivotal downstream genes in this pathway, including *cyclin D1* (*CCND1*) and *cMYC*, which are well known for their involvement in cell cycle progression and proliferation of cancer cells. When we treated two HCC cells and two colon cancer cells with siRNA for SMYD2 (siSMYD2), significant downregulation of both *CCND1* and *cMYC* was observed in concordance with the decrease of nuclear β-catenin in all of these four cell lines (Figure 3A). The data for other downregulated genes after the treatment with siSMYD2 for these cell lines are shown in Supplementary Figure 6. We also found significantly lower level of *CCND1* transcript in 293T cells in which K133A-substituted β-catenin and SMYD2 were co-transfected, compared those co-transfected with WT-β-catenin and SMYD2 (Supplementary Figure 7). These results strongly imply that β-catenin methylation by SMYD2 is essential for nuclear translocation of β-catenin and subsequent activation of the Wnt/β-catenin/TCF pathway.

### SMYD2-mediated methylation is critical for β-catenin nuclear transportation by FOXM1 and cancer cell growth

Because FOXM1 is suggested to play an important role in translocation of β-catenin into the nucleus [17], we examined possible effect of β-catenin methylation on the interactions of these two proteins by a co-immunoprecipitation experiment. Western blot analysis confirmed the interaction between WT-β-catenin and FOXM1 (co-immunoprecipitation of FOXM1 with β-catenin), but the interaction of K133A-substituted β-catenin and FOXM1 was almost completely diminished (Figure 3B), indicating that methylation at K133 in β-catenin is required for its interaction with FOXM1. Since BCL9 is also reported to make a complex with β-catenin and regulates β-catenin transcriptional activity, we also examined co-immunoprecipitation of BCL9 with β-catenin and found the loss of interaction between BCL9 and β-catenin when lysine 133 was substituted to alanine (Figure 3B). However, because BCL9 is mainly localized in the nucleus, the lack of interaction might simply reflect the absence of K133A-substituted β-catenin in the nucleus under this experimental condition. As shown in Figure 3B, western blot analysis indicated K133A-substituted β-catenin was not methylated by SMYD2 and lost the interaction with FOXM1. However, the K133A-substituted β-catenin and SMYD2 could be co-immunoprecipitated, suggesting that this lysine-alanine substitution did not cause the significant structural change in β-catenin protein and maintained the interaction with SMYD2, but solely affected the methylation by SMYD2. Furthermore, we also found the interaction of β-catenin and α-catenin was enhanced in the condition that β-catenin K133 was substituted with an alanine residue as shown in Figure 3B. To further understand this enhanced interaction, we performed a structural analysis of β-catenin. As shown in Figure 3C, a region including the methylation site of β-catenin is indicated to be important for the interaction with α-catenin. Thus, the methylation of β-catenin at K133 might cause the dissociation of β-catenin from α-catenin and increase the free form of β-catenin in the cytoplasm, resulting in enhancement of the β-catenin-FOXM1 interaction.

**Figure 4:**
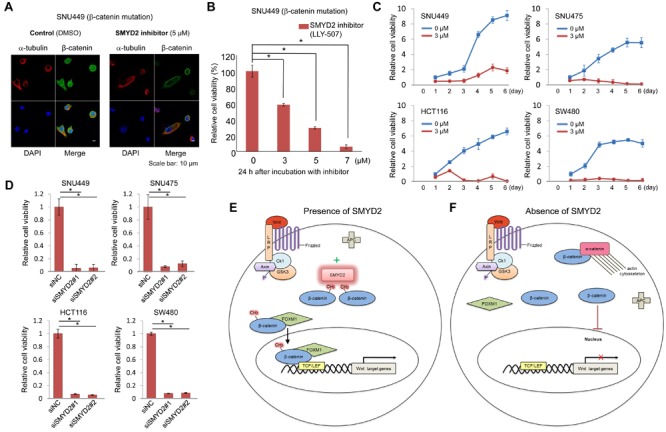
SMYD2-mediated β-catenin methylation is critical for cancer cell growth **A.** Significant decrease of nuclear β-catenin after treatment with SMYD2 inhibitor (LLY-507) was observed by ICC. SNU449 cells were treated with 5 μM of LLY-507 (a SMYD2-specific inhibitor) or DMSO, and incubated for 48 h. Cells were then fixed and stained with an anti-β-catenin antibody (rabbit, Alexa Fluor^®^ 488, green), an anti-α -tubulin antibody (mouse, Alexa Fluor^®^ 594, red) and 4′,6′-diamidine-2′-phenylindole dihydrochloride (DAPI, blue). **B.** The effect on the viability of SNU449 cells by LLY-507. LLY-507 was added to the culture medium of SNU449 cells at different concentrations (0, 3, 5, or 7 μM). **C.** Cell growth curves of four cancer cell lines under the treatment of LLY-507 at 0 and 3 μM for six days. Significant growth-suppressive effects on cancer cells were observed in the cells treated with inhibitors in comparison of the control group. **D.** Cell viability assay of four cancer cell lines treated with siRNA. Significant decrease of the number of viable cells was observed in cells treated with siSMYD2-#1 and #2, compared with those treated with siNC after 5-day (SNU449 and SNU475) or 7-day (HCT116 and SW480) of the treatment. The asterisks indicate *p* value of < 0.005 compared with the control using an unpaired Student's *t*-test (two groups). Error bars indicate values of one standard deviation (*n* = 3). (E, F) Schematic presentation of the proposed mechanism of the Wnt signaling pathway in the presence **E.** or absence **F.** of SMYD2.

To further verify the biological and clinical significance of K133 methylation, we treated SNU449 HCC cells with a SMYD2-specific inhibitor, LLY-507 [18]. After 20 h of incubation with 5 μM concentration of the inhibitor, we observed the drastic decrease of nuclear β-catenin in more than 70 % of SNU449 cells (Figure 4A). As shown in Figure 4B, the number of viable cells was significantly decreased with the inhibitor treatment in a dose-dependent manner, implying that an inhibitor(s) targeting SMYD2 could suppress SMYD2-mediated β-catenin methylation, reduce β-catenin nuclear translocation, and subsequently induce cancer cell death. Figure 4C revealed the growth suppressive effects of SMYD2-specific inhibitor (at 3 μM concentration) on two HCC cells (SNU449 and SNU475) and two colon cancer cells (HCT116 and SW480). Concordantly, treatment with siSMYD2 showed the significant growth suppressive effect on the four cancer cell lines compared with siNC (Figure 4D), implying that targeting SMYD2 could suppress SMYD2-mediated β-catenin methylation and induce the death of cancer cells harboring mutations in β-catenin or those in the β-catenin degradation machinery genes. We then further investigated if this SMYD2 mediated methylation of β-catenin could play important role in cell proliferation. We transfected either of Flag-Mock, Flag-WT-β-catenin and Flag-K133A-β-catenin plasmids into SNU449 and SNU475 cells. As shown in Supplementary Figure 8, significant decrease of the number of cells was observed in cells transfected with Flag-K133A-β-catenin, while increase of the cell number was observed in cells with Flag-WT-β-catenin expressed, compared to the control cells (Flag-Mock), indicating the dominant negative effect of K133A-β-catenin probably due to the inhibition of interaction between WT-β-catenin and SMYD2. In addition, the treatment of SMYD2-specific inhibitor (LLY-507) in the cells transfected with Flag-WT-β-catenin completely diminished the growth promoting effect of WT-β-catenin, compared to the control cells (DMSO). These results further support that the growth promoting effect by WT-β-catenin requires the SMYD2-dependent monomethylation on this protein.

## DISCUSSION

We have demonstrated (1) that SMYD2 methylates Lys133 of β-catenin, (2) that methylated β-catenin can bind to FOXM1, and then (3) that β-catenin can be translocated into the nucleus with FOXM1. Concordantly, Lys133Ala-substituted β-catenin could not be methylated (although it could interact with SMYD2) and could not enter into the nucleus. Knockdown of SMYD2 abolished β-catenin methylation and nuclear localization of β-catenin through the loss of the interaction with FOXM1, resulted in the downregulation of the Wnt/β-catenin/TCF downstream genes such as *c-myc* and *cyclin D*, and subsequently induced cancer cell death. Furthermore, treatment of cancer cells with the SMYD2-specific inhibitor also abolished the nuclear localization of the β-catenin. These lines of evidences imply that SMYD2-medicated β-catenin methylation is critically essential for β-catenin nuclear translocation and the subsequent activation of the Wnt/β-catenin/TCF pathway.

It has been known for many years that dysfunction of β-catenin degradation machinery or β-catenin activating-mutations cause the stabilization and nuclear accumulation of β-catenin in many types of human cancer including colon cancer and hepatocellular carcinoma. The presence of nuclear β-catenin accumulation is considered as a hallmark of the activation of the Wnt/β-catenin/TCF pathway. Somehow, we thought that the stabilization of β-catenin in the cytoplasm could spontaneously cause the translocation of β-catenin into the nucleus and the accumulation of a large amount of β-catenin in the nucleus, and activate the Wnt/β-catenin/TCF pathway. However, our results clearly imply that methylation of β-catenin by SMYD2 is critically important for its nuclear translocation and the activation of the Wnt/β-catenin/TCF pathway. The fact that K133A-substituted β-catenin, which lacked a methylation site, could interact with SMYD2, but lost the interaction with FOXM1, indicated the significance of K133 methylation for the interaction with FOXM1, and the subsequent nuclear translocation of β-catenin with FOXM1. In addition, a region including the methylation site of β-catenin is suggested to be important for the interaction with α-catenin (Figure 3C). Hence, β-catenin K133 methylation might cause the dissociation of β-catenin from α-catenin and enhance the interaction with FOXM1. We summarize this possible mechanism showing the significance of SMYD2-medicated β-catenin in Figure 4E and 4F; when one of the molecules involved in the β-catenin degradation machinery (APC, Axin, or GSK3β etc.) is mutated or β-catenin itself is activated by somatic mutation, β-catenin is stabilized, but cannot enter into the nucleus in the absence of SMYD2 (Figure 4F). However, when SMYD2 is transactivated in cancer cells, stabilized β-catenin is methylated by SMYD2, binds to FOXM1, enters into the nucleus with FOXM1, and then activates downstream genes in the Wnt/β-catenin/TCF pathway.

Although we need to further elucidate the detailed mechanism using an animal model, our data strongly support the significance of SMYD2-medicated β-catenin methylation for the Wnt/β-catenin/TCF pathway activation. Hence, our results should shed light on a novel and critical process for the activation of the Wnt/β-catenin/TCF pathway. Since SMYD2 is overexpressed in a large proportion of human cancers including colon cancer, breast cancer, and hepatocellular carcinoma, development of novel anti-cancer drugs targeting SMYD2 should be a very effective way to treat various types of human cancer in which the Wnt/β-catenin/TCF pathway is activated.

## MATERIALS AND METHODS

### Cell lines

293T, SNU449, SNU475, HCT116 and SW480 cell lines were obtained from American Type Culture Collection ATCC, tested and authenticated by DNA profiling for polymorphic short tandem repeat (STR) markers (see Certificated information of cell lines in Supplementary Table 1). All cells were grown in the monolayers in appropriate media supplemented with 10% fetal bovine serum and 1% antibiotic/antimycotic solution (Life Technologies): Dulbecco's modified Eagle's medium (D-MEM) for 293T cells; RPMI 1640 Medium for SNU449 and SNU475; McCoy's 5A medium for HCT116 cells; Leibovitz's L-15 Medium for SW480 cells. All cells were maintained at 37°C in humid air with 5% CO_2_ condition except for SW480 cells (without CO_2_).

### Antibodies

An anti-K133 monomethylated β-catenin antibody was generated in rabbit by immunization with a synthetic peptide. The amino acid sequences of modified and unmodified peptides are shown in Supplementary Table 2. The following primary antibodies were used for western blot (WB) and immunocytochemical (ICC) analysis: anti-K133 monomethylated β-catenin antibody (1:500 dilution for WB and 1:200 dilution for ICC); anti-FLAG tag (M2, mouse, #F3165; Sigma-Aldrich, St. Louis, MO; 1:2000 dilution for WB and 1:500 dilution for ICC), anti-SMYD2 (D14H7, rabbit, #9734; Cell Signaling Technology; 1:500 dilution for WB and 1:100 dilution for ICC), anti-β-catenin (carboxy-terminal antigen, rabbit, #9587; Cell Signaling Technology; 1:500 dilution for WB and 1:100 dilution for ICC), anti-α-tubulin (DM1A, mouse; CALBIOCHEM, Billerica, MA; 1:500 dilution for WB and 1:100 dilution for ICC), anti-histone H3 (rabbit, ab1791; Abcam; 1:500 dilution for WB), anti-HA (3F10, rat, #11867423001; Roche;1:2000 dilution for WB and 1:500 dilution for ICC ), anti-FOXM1 (D12D5, Rabbit, #5436S; Cell Signaling Technology; 1:500 dilution for WB and 1:100 dilution for ICC), anti-BCL9 (rabbit, #15096S, Cell Signaling Technology, 1:500 dilution for WB).

### *In vitro* methyltransferase assay

*In vitro* methyltransferase assays were performed as described previously [19, 20]. Briefly, recombinant GST-WT-β-catenin protein (#12-537; Millipore) was incubated with recombinant His-SMYD2 protein (in-house) and 2 μCi S-adenosyl-L- [methyl-^3^H]-methionine (Perkin Elmer, Waltham, MA) in a mixture of methylase activity buffer (50 mM Tris-HCl at pH 8.8, 10 mM DTT and 10 mM MgCl_2_) for 2 h at 30°C. After denaturing, samples were separated by SDS-PAGE, blotted to PVDF membrane and visualized by MemCode Reversible Stain (Thermo Fisher Scientific, Waltham, MA) and fluorography.

### Mass spectrometry

The bands corresponding to β-catenin were exacted from the gel, and digested with sequencing grade TPCK-trypsin (Worthington Biochemical, Lakewood, NJ) at 37°C for 12 h. The digestion mixture was analyzed by nano liquid chromatography-tandem mass spectrometry (nLC-MS/MS) using Q-Exactive mass spectrometer (Thermo Fisher Scientific, San Jose, CA). The peptides were separated using nano ESI spray column on an NTCC analytical column (C18, φ0.075 × 100 mm, 3 μm, Nikkyo Technos, Tokyo, Japan) at a flow rate 300 nL/min. The mass spectrometer was operated in the positive-ion mode, and the spectra were acquired in a data-dependent TOP 5 MS/MS mode. The MS/MS spectra were searched against the in-house database using local MASCOT server (version 2.5; Matrix Sciences, London, United Kingdom).

### Cloning of expression vectors

Full-length β-catenin cDNA was cloned into the pCAGGS-n3FC (FLAG tag) vector. Full-length SMYD2 cDNA was cloned into the pCAGGS-nHC (HA tag) vector. Substitution of β-catenin at lysine 133 to alanine was introduced using Q5 site direct mutagenesis kit (New England Biolabs) according to the manufacture's protocol. Expression vectors were transfected into cells using FuGENE HD (Promega, Fitchburg, WI) according to manufacturer's protocols.

### Western blotting

Samples were prepared from the cells lysed with CelLytic^TM^ M cell lysis reagent (Sigma-Aldrich, St. Louis, MO) supplemented with a protease inhibitor (cOmplete^TM^ protease inhibitor cocktail, Roche Applied Science). Whole cell lysates, fractionated cell lysates or immunoprecipitated samples were separated by SDS-PAGE and blotted to nitrocellulose membrane. Nitrocellulose membrane was firstly incubated with each primary antibody as described in the above Antibodies section, and then protein bands were detected by incubating secondary antibody: horseradish peroxidase (HRP)-conjugated antibodies (GE Healthcare, Little Chalfont, UK) and visualizing with enhanced chemiluminescence (Thermo Fisher Scientific) and Prime enhanced chemiluminescence (GE Healthcare, GE Healthcare, Little Chalfont, UK). Nuclear and cytoplasmic fractions were prepared from SNU449 and SNU475 cells 72 h after siRNA transfection and were prepared from 293T cells 48 h after transfection of an indicated expression vector(s). All the samples were fractionated by using NE-PER Nuclear and Cytoplasmic Extraction Reagents (Thermo Fisher Scientific) according to the manufacture's protocol.

### Immunoprecipitation

For whole-cell extract, transfected 293T cells were lysed by CelLytic^TM^ M cell lysis reagent (Sigma-Aldrich, St. Louis, MO) and a subsequent immunoprecipitation reaction was conducted. Before lysing the cells, a protease inhibitor (cOmplete^TM^ protease inhibitor cocktail, Roche Applied Science) was added to cell lysis reagent under instructions of the manufacture. For fractionated cytoplasmic and nuclear components, immunoprecipitation reaction was conducted directly after cell fractionation. In an immunoprecipitation reaction, 300 μg of cell extract were incubated with 30 μL of anti-FLAG M2 affinity gel (Sigma-Aldrich, #A2220), which is a purified monoclonal anti-FLAG antibody covalently attached to agarose beads. After the beads were washed 3 times in 1 ml of TBS buffer (pH 7.6), proteins that bound to the beads were eluted by boiling in Lane Marker Reducing Sample Buffer (Thermo Fisher Scientific).

### Small interfering RNA transfection

siNegative control (siNC) from Sigma-Aldrich, a mixture of three different oligonucleotide duplexes was used as a control siRNA. Two siRNA oligonucleotide duplexes were purchased from Sigma-Aldrich for targeting SMYD2 transcripts, and named as siSMYD2#1 and -#2. The siRNA sequences are described in Supplementary Table 3. siRNA duplexes were transfected with Lipofectamine RNAi max (Life Technologies) according to the instruction of manufacture.

### Immunocytochemistry

Cells were fixed in 4% paraformaldehyde in 0.1 M phosphate buffer at 4°C for 1 h, permeabilized in 0.1% Triton X-100 (Sigma-Aldrich) for 3 min at room temperature and blocked with 3% BSA for 1 h at room temperature. Fixed cells were incubated with anti-α-tubulin, anti-β-catenin, anti-FLAG and anti-HA antibodies overnight at 4°C followed by incubation with Alexa Fluor-conjugated secondary antibody (Life Technologies, Carlsbad, CA), and observed using Leica confocal microscopy (SP5 tandem Scanner Spectral 2-Photon Confocal). Signal intensity (mean value of selected area) of exogenously expressed β-catenin was quantified using ImageJ software (https://imagej.nih.gov/ij).

### Real-time RT-PCR

Specific primers for human *GAPDH* (housekeeping gene), *SMYD2, CCND1*, *cMYC, c-JUN, VEGF, Axin2* and *claudin-1* were designed (primer sequences in Supplementary Table 4). PCR reactions were performed using ViiA™ 7 real-time PCR system (Thermo Fisher Scientific) following the protocol of the manufacture. mRNA levels were normalized to *GAPDH* expression level.

### Cell viability

Cancer cells were seeded into 96-well flat-bottom plates (BD Falcon) at 4000 cells per well, and treated with a SMYD2-specific inhibitor, LLY-507 (≥97%, HPLC, #SML1279, Sigma-Aldrich), at concentration of 0, 3, 5, or 7 μM, and cultured at 37°C under 5% CO_2_ for 24 h. We also examined growth suppressive effects of LLY-507 at 3 μM concentration in four cancer cell lines, compared without the compound. The growth suppressive effects by knockdown of SMYD2 were assessed after 5 days (rapidly-growing cancer cell lines, SNU449 and SNU475) or 7 days (slowly-growing cancer cell lines, HCT116 and SW480) of transfection with siRNAs. The Cell counting kit-8 (Dojindo Molecular Technologies, Inc., Kumamoto, Japan) was used for methyl thiazolyl tetrazolium reaction. After reaction for 2 h, the numbers of cells were counted in a microplate reader at 450 nm.

### Statistical analysis

Values were presented as the means plus or minus one standard deviation. Statistical analyses were performed using unpaired Student's *t*-test (two groups). Significant difference between groups was noted when *P*-value was < 0.05.

## SUPPLEMENTARY MATERIALS FIGURES AND TABLES


