# Supplementary material for: Critical roles of SMYD2-mediated β-catenin methylation for nuclear translocation and activation of Wnt signaling
Source: Oncotarget. 2017 Jul 27;8(34):55837–47. doi: 10.18632/oncotarget.19646 (PMC5593527; doi:10.18632/oncotarget.19646)
Supplement: Supplementary file 1 [file oncotarget-08-55837-s001.pdf]

Critical roles of SMYD2-mediated β-catenin methylation for nuclear translocation and activation of Wnt signaling

SUPPLEMENTARY MATERIALS

a

b

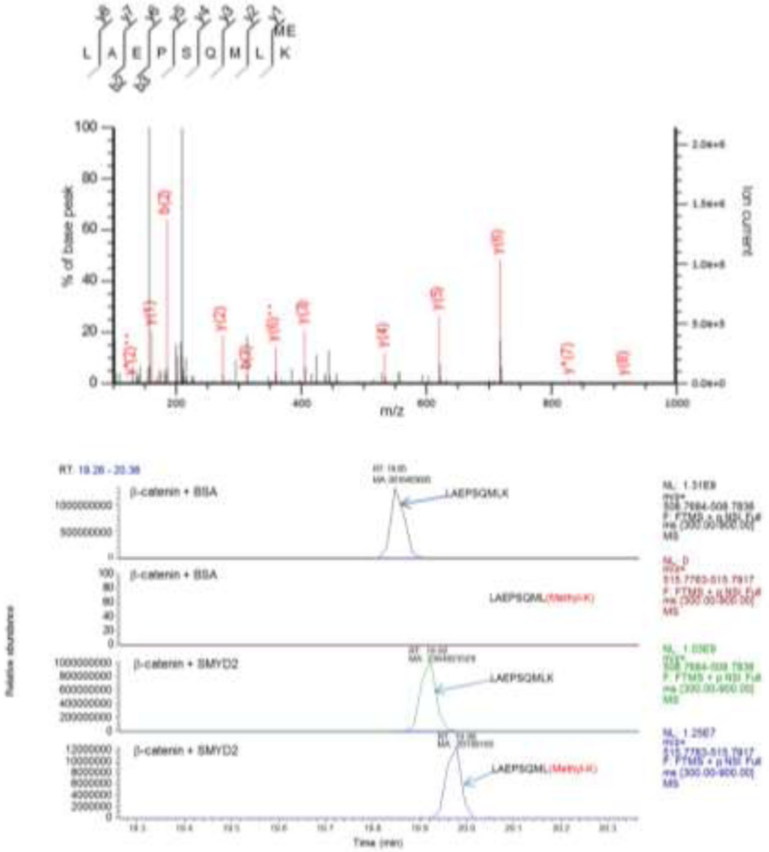

c

#	b	b^{++}	b^{*}	b^{*++}	b^{0}	b^{0++}	Seq.	y	y^{++}	y^{*}	y^{*++}	y^{0}	y^{0++}	#
1	114.09	57.55					L							9
2	185.13	93.07					A	917.48	459.24	900.45	450.73	899.47	450.24	8
3	314.17	157.59			296.16	148.58	E	846.44	423.72	829.41	415.21	828.43	414.72	7
4	411.22	206.12			393.21	197.11	P	717.40	359.20	700.37	350.69	699.39	350.20	6
5	498.26	249.63			480.25	240.63	S	620.34	310.68	603.32	302.16	602.33	301.67	5
6	626.31	313.66	609.29	305.15	608.30	304.66	Q	533.31	267.16	516.29	258.65			4
7	757.35	379.18	740.33	370.67	739.34	370.18	M	405.25	203.13	388.23	194.62			3
8	870.44	435.72	853.41	427.21	852.43	426.72	L	274.21	137.61	257.19	129.10			2
9							K	161.13	81.07	144.10	72.55			1

Supplementary Figure 1: Summary of LC-MS/MS data. (**a**) The LC-MS/MS spectrum corresponding to the monomethylated peptide corresponding to codon 125–133 of β-catenin which was *in vitro* methylated by recombinant SMYD2. The 14-Da increase of the Lysine 133 residue was observed by all y ions. (**b**) Selected full MS ion chromatograms of modified and monomethylated β-catenin 125-133 peptides in the LC-MS/MS. (**c**) The theoretical values of MS/MS fragment ions of the Lysine 133 monomethylated β-catenin 125–133 peptide are summarized in the table. The abbrevations of fragment ion types were indicated by the MASCOT program (http://www.matrixscience.com/help/fragmentation-help.html). The observed ions in extended data figure 1a, are indicated in red letters.

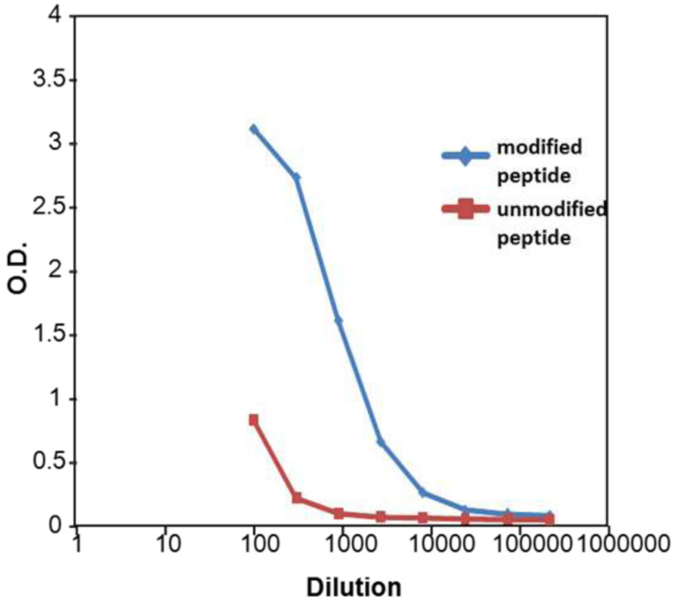

Supplementary Figure 2: Determination of the titer and specificity of the anti-monomethylated K133 β-catenin antibody analyzed by ELISA. The blue line represents that the anti-monomethylated K133 β-catenin antibody specifically and quantitatively recognizes the K133-monomethylated β-catenin 125-133 peptide, compared with the red line representing the activity against the unmodified β-catenin 125-133 peptide. The amino acid sequences of modified and unmodified peptides are shown in Supplementary Table 2. Error bars indicate values of one standard deviation (n=3).

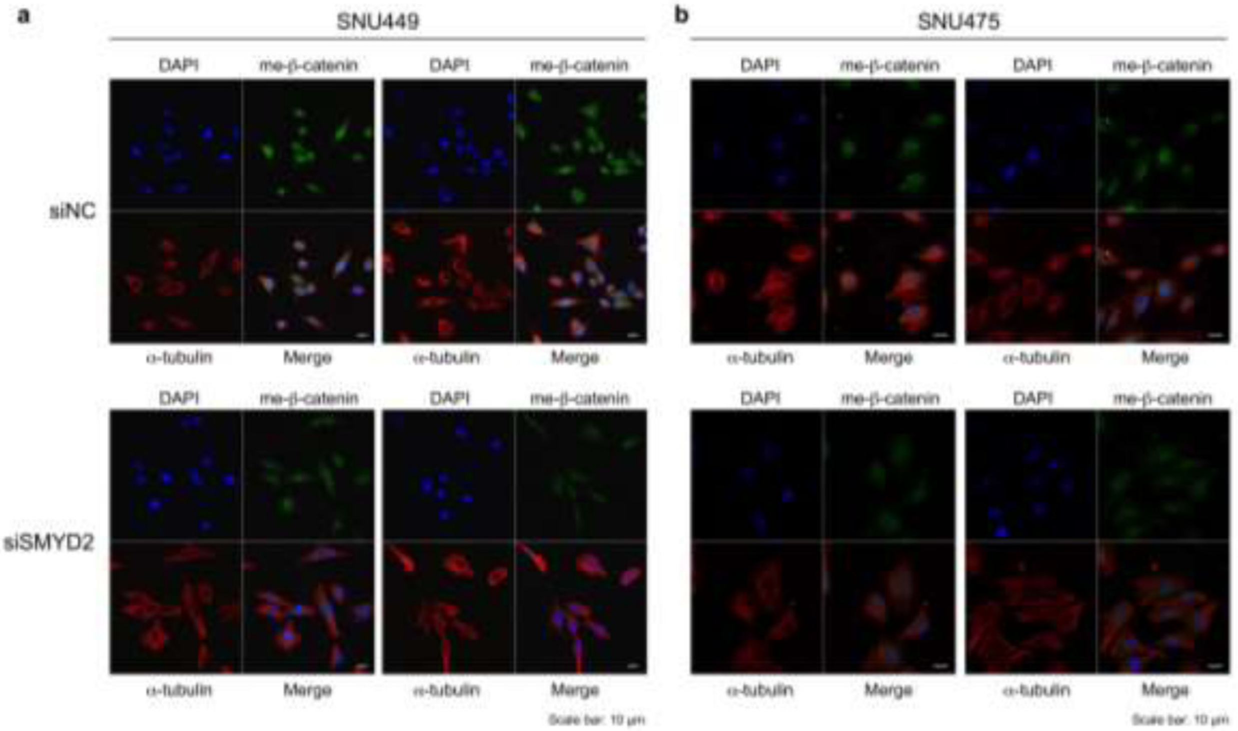

Supplementary Figure 3: Knockdown of SMYD2 attenuates monomethylation (meK133) of β-catenin in nucleus. Significant reduction of mono-methylated (meK133) β-catenin was observed in the nuclei of SNU449 (**a**) and SNU475 (**b**) cells by knockdown of SMYD2. Cells were transfected with siNC (control) or siSMYD2 (SMYD2#2). After 48h-incubation with siRNAs, cells were fixed with 4% paraformaldehyde, and stained with an anti-α-tubulin antibody (Alexa Fluor® 594, red), anti-meK133-β-catenin antibody (Alexa Fluor® 488, green) and 4',6'-diamidine-2'-phenylindole dihydrochloride (DAPI, blue).

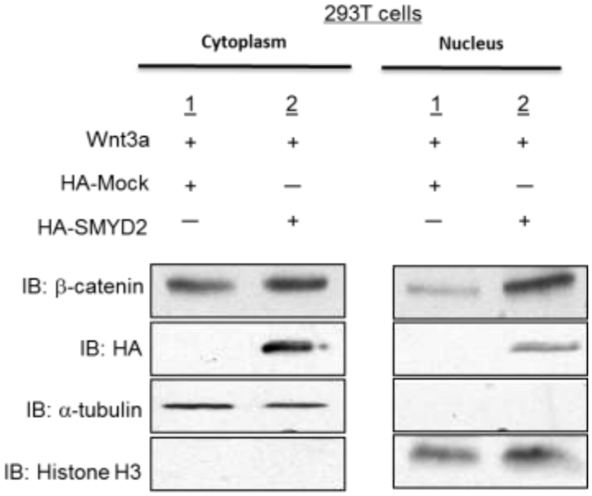

Supplementary Figure 4: SMYD2-mediated β-catenin methylation promotes nuclear translocation of β-catenin in 293T cells. Drastic increase of β-catenin in the nucleus was observed by western blot analysis. 293T cells were treated with 40 ng/mL of Wnt3a for 2 h, then the cells were transfected with HA-Mock or HA-SMYD2, and further cultured for 48 h. Samples were immunoblotted with anti-β-catenin, anti-HA, anti-α-tubulin and anti-histone-H3 antibodies.

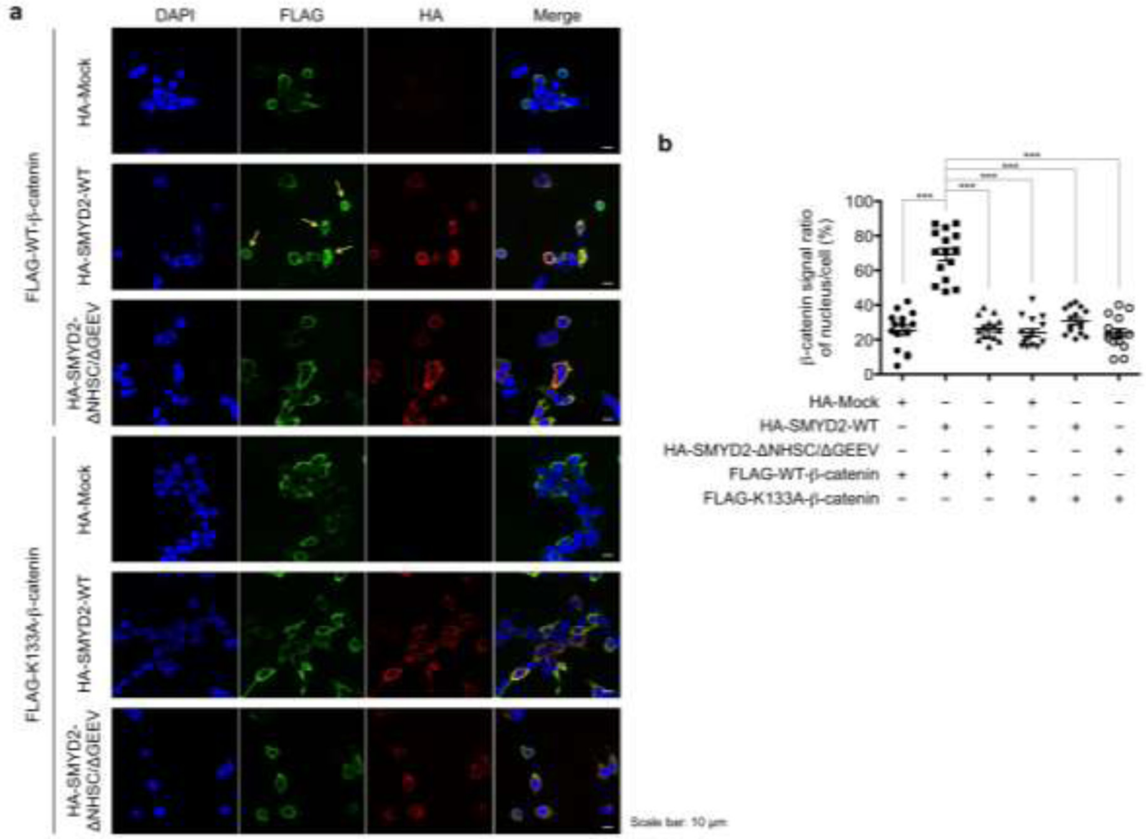

Supplementary Figure 5: Nuclear translocation of β-catenin is dependent on SMYD2 enzyme activity and monomethylation at a K133 residue. (**a**) 293T cells were treated with 40 ng/mL of Wnt3a for 2 h, then the cells were co-transfected with HA-Mock, HA-SMYD2-WT, or HA-SMYD2 enzyme-dead (ΔNHSC/ΔGEEV), combined with FLAG-WT-β-catenin or FLAG-K133A-β-catenin. After 48 h of additional culture, cells were fixed with 4% paraformaldehyde, and stained with an anti-FLAG antibody (Alexa Fluor® 488, green), anti-HA antibody (Alexa Fluor® 594, red) and 4',6'-diamidine-2'-phenylindole dihydrochloride (DAPI, blue). Arrows indicate nuclear localization of FLAG-WT-β-catenin. (**b**) Signal intensity of β-catenin (green) in the nucleus area as well as the whole cell area in 293T cells was quantified. Then, the β-catenin signal ratio of nucleus/whole cell was graphed after quantification of 15 cells per each group. The asterisks (***) indicate p-value of < 0.001 compared with 293T cells co-transfected with HA-SMYD2-WT and FLAG-WT-β-catenin, using an unpaired Student's t-test (two groups).

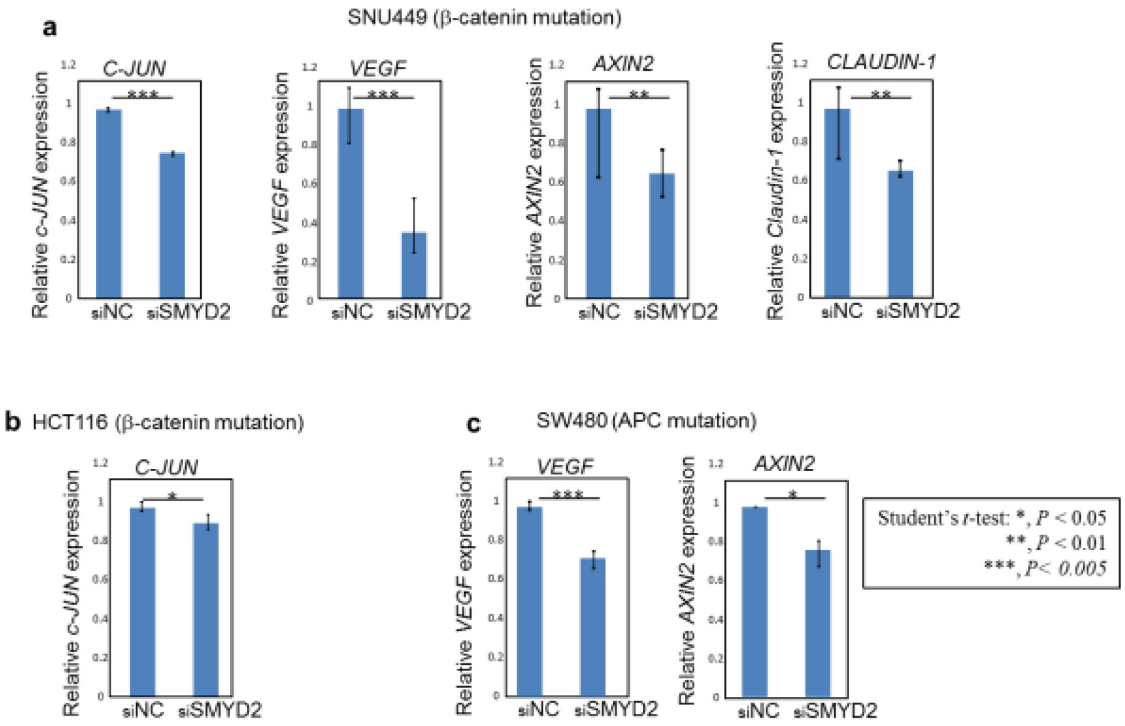

Supplementary Figure 6: SMYD2-mediated β-catenin methylation is required for β-catenin nuclear translocation and expression of Wnt downstream genes of each cell lines. (**a**) SMYD2 knockdown attenuated transcriptional levels of the Wnt pathway downstream genes: *C-JUN, VEGF, AXIN2* and *CLAUDIN-1* in SNU449 cells (**b**) *C-JUN* in HCT116 (**c**) *VEGF* and *AXIN2* in SW480 cells. Cells were transfected with siNC or siSMYD2 (siSMYD2#1). After 48 h incubation, RNAs were prepared from these cells and transcriptional levels of downstream genes were measured by quantitative RT-PCR. Statistical analyses were performed using unpaired Student's t-test (two groups). The asterisks indicate statistical significance; *, **, and *** indicate p-value of <0.05, <0.01 and <0.005, respectively, compared to the corresponding value of the siNC (control) group. Error bars indicate values of one standard deviation (n=3).

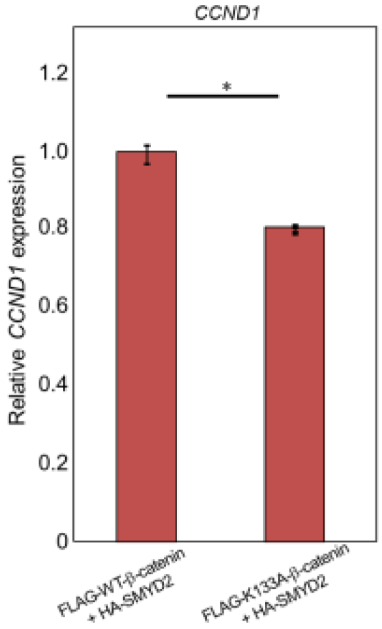

Supplementary Figure 7: Transcriptional level of Wnt pathway downstream gene, CCND1, was lower in 293T cells where FLAG-K133A-substituted β-catenin was co-transfected with HA-SMYD2, than those where FLAG-WT-β-catenin was co-transfected with HA-SMYD2. After 48 h of incubation, RNAs were extracted from cells and transcriptional levels of *CCND1* were measured by quantitative RT-PCR. Statistical analysis was performed using unpaired Student's t-test (two groups). The asterisk indicates statistical significance at p-value of <0.05, compared to the corresponding value of the FLAG-WT-β-catenin + HA-SMYD2 group. Error bars indicate values of one standard deviation (n=3).

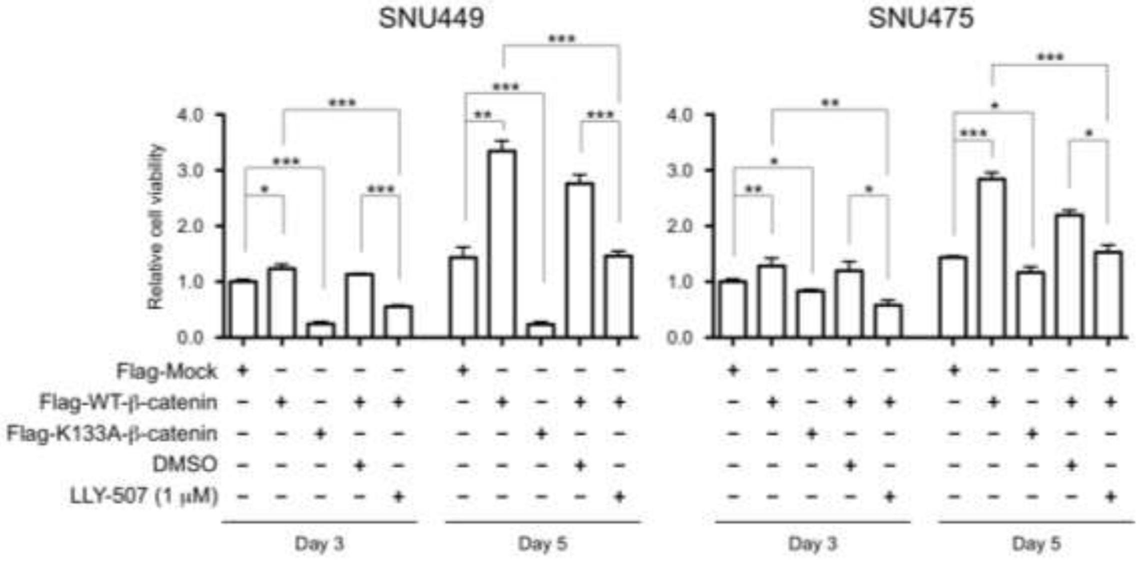

Supplementary Figure 8: SMYD2-mediated methylation of β-catenin plays significant roles in cell proliferation. SNU449 and SNU475 cells were transfected with Flag-Mock, Flag-WT-β-catenin or Flag-K133A-β-catenin. DMSO (control) or SMYD2-specific inhibitor, LLY-507 (1uM) was added in the cells transfected with Flag-WT-β-catenin. Cell viability assays were performed at day 3 and day 5. Significant increase of the number of viable cells was observed in cells transfected with Flag-WT-β-catenin plasmid while significant decrease of the number of viable cells was observed in cells transfected with Flag-K133A-β-catenin plasmid, compared with those transfected by Flag-Mock at day 5. In addition, growth-promoting effect of WT-β-catenin was completely diminished by addition of LLY-507. Statistical analyses were performed using unpaired Student's t-test (two groups). The asterisks indicate statistical significance; *, **, and *** indicate p-value of <0.05, <0.01 and <0.005, respectively. Error bars indicate values of one standard deviation (n=3).

Supplementary Table 1: Certificated information of cell lines

Name	Certification institution	Tested method	DNA profile
293T	ATCC	STR	Amelogenin: X CSF1PO: 11, 12 D13S317: 12, 14 D16S539: 9, 13 D5S818: 8, 9 D7S820: 11 THO1: 7, 9.3 TPOX: 11 vWA: 16, 18, 19
SNU475	ATCC	STR	Amelogenin: X,Y CSF1PO: 11,12 D13S317: 8, 11 D16S539: 12 D5S818: 10, 13 D7S820: 7, 12 THO1: 7, 9 TPOX: 8, 9 vWA: 14
SNU449	ATCC	STR	Amelogenin: X,Y CSF1PO: 7, 10 D13S317: 10, 12 D16S539: 11, 13 D5S818: 10, 11 D7S820: 11, 12 THO1: 6, 9 TPOX: 11 vWA: 14, 16
HCT116	ATCC	STR	Amelogenin: X CSF1PO: 13, 14 D13S317: 12 D16S539: 13 D5S818: 13 D7S820: 8 THO1: 8 TPOX: 11 vWA: 16
SW480	ATCC	STR	Amelogenin: X,Y CSF1PO: 11 D13S317: 9 D16S539: 9 D5S818: 10 D7S820: 8, 13 THO1: 6, 9 TPOX: 11 vWA: 14, 16

ATCC; American Type Culture Collection
STR; Short Tandem Repeat

Supplementary Table 2: Sequence of modified and unmodified peptide

Peptide Name	Amino Acid Sequence
K133A-β-catenin (me-β-catenin, modified)	Ac-AEP SQM LK(Me)H AVVC-NH2
WT-β-catenin (unmodified)	Ac-AEP SQM LKH AVV C-NH2

Supplementary Table 3: siRNA sequences

siRNA name		Sequence	
siNegative control (cocktail)	Target #1	Sense	AUCCGCGCGAUAGUACGUA
		Antisense	UACGUACUAUCGCGCGGAU
	Target #2	Sense	UUACGCGUAGCGUAAUACG
		Antisense	CGUAUUACGCUACGCGUAA
	Target #3	Sense	UAUUCGCGCGUAUAGCGGU
		Antisense	ACCGCUAUACGCGCGAAUA
siSMYD2#1		Sense	GAUUUGAUUCAGAGUGACA
		Antisense	UGUCACUCUGAAUCAAAUC
siSMYD2#2		Sense	GAAAUGACCGGUUAAGAGA
		Antisense	UCUCUUAACCGGUCAUUUC

Supplementary Table 4: Primer sequences for quantitative RT-PCR

Gene name	Primer sequence (5'-3')
GAPDH(housekeeping gene)-f	GCAAATTCCATGGCACCGTC
GAPDH(housekeeping gene)-r	TCGCCCCACTTGATTTTGG
SMYD2-f<br>*SMYD2-r*<br>*CCND1-f*	ATCTCCTGTACCCAACGGAAGATAG<br>CACCTTGGCCTTATCCTTGTCCTTG<br>CTGTGCATCTACACCGACAACTC
CCND1-r	ACTTGAGCTTGTTCACCAGGAGC
cMYC-f	CTCGGATTCTCTGCTCTCCTCGAC
cMYC-r	TCTTGTTCCTCCTCAGAGTCGCTG
c-JUN-f	CCAAAGGATAGTGCGATGTTT
c-JUN-r	CTGTCCCTCTCCACTGCAAC
VEGF-f	CTACCTCCACCATGCCAAGT
VEGF-r	GCAGTAGCTGCGCTGATAGA
Axin2-f	ACAACAGCATTGTCTCCAAGCAGC
Axin2-r	GCGCCTGGTCAAACATGATGGAAT
Claudin-1-f	GCGCGATATTTCTTCTTGCAGG
Claudin-1-r	TTCGTACCTGGCATTGACTGG

Supplementary raw data (all electrophoresis results)

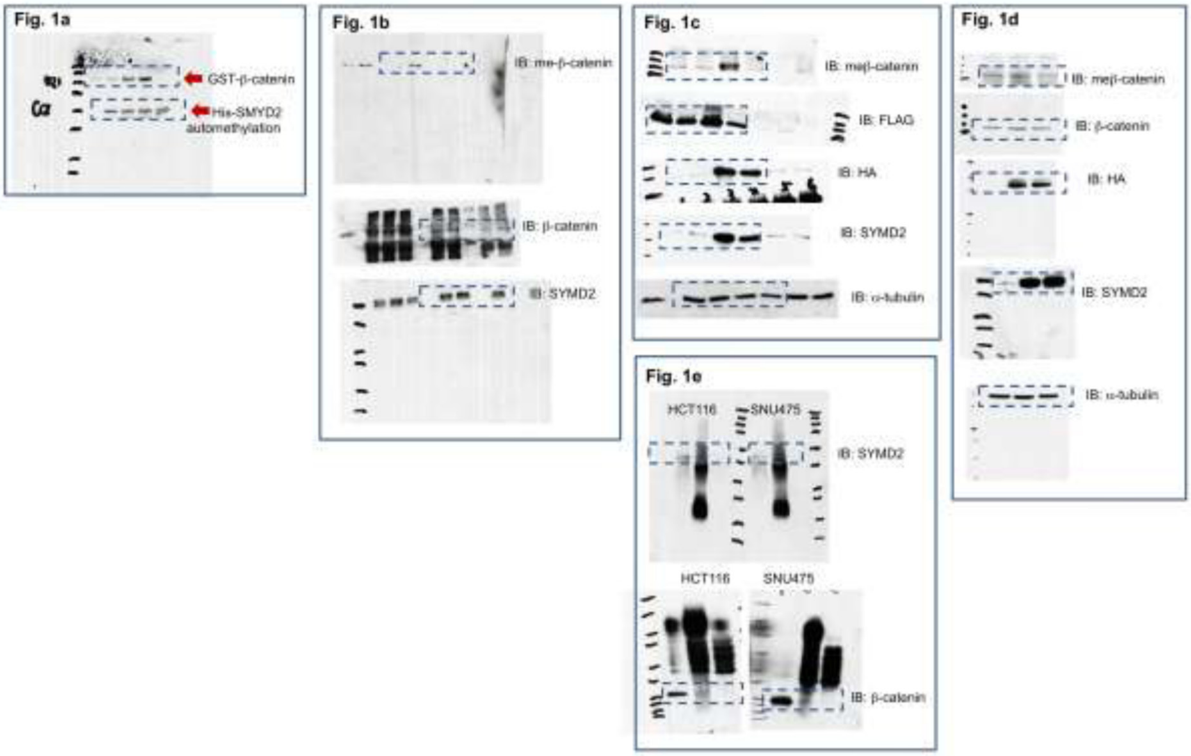

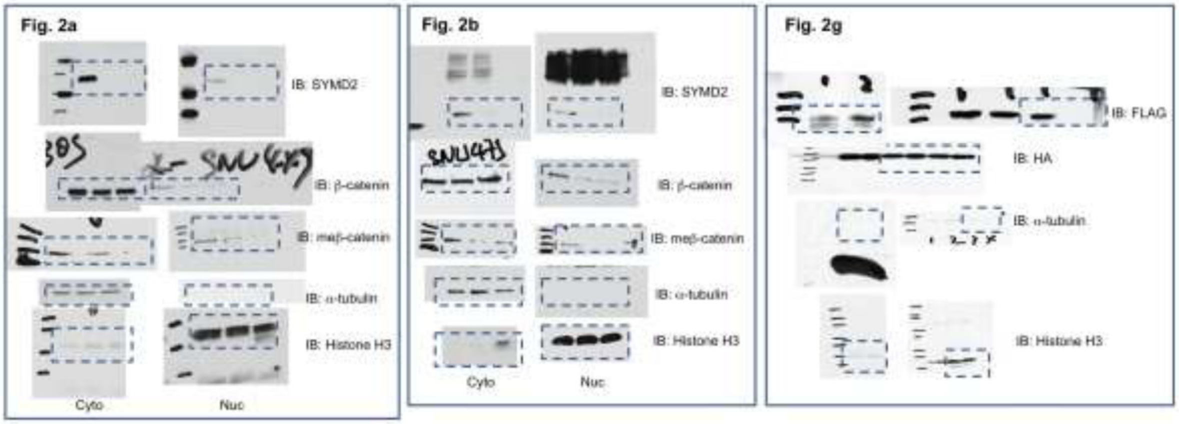

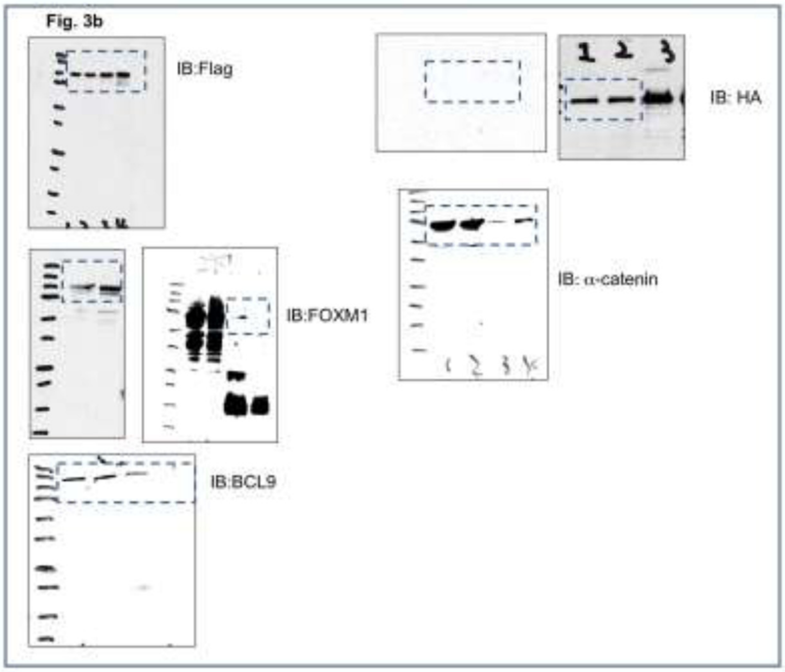
Fig. 3b
IB:Flag
IB: HA
1 2 3
IB: α-catenin
IB:FOXM1
IB:BCL9
